# Insight of transcriptional regulators reveals the tolerance mechanism of carpet-grass (*Axonopus compressus*) against drought

**DOI:** 10.1186/s12870-021-02844-7

**Published:** 2021-02-02

**Authors:** Mohsin Nawaz, Liao Li, Farrukh Azeem, Samina Shabbir, Ali Zohaib, Umair Ashraf, Hubiao Yang, Zhiyong Wang

**Affiliations:** 1 Key Laboratory of Genetics and Germplasm Innovation of Tropical Special Forest Trees and Ornamental Plants, Ministry of Education, College of Forestry and College of Tropical Crops, Hainan University, Haikou, 570228 People’s Republic of China; 2 Department of Bioinformatics and Biotechnology, Govt. College University, Faisalabad, Pakistan; 3 Key Laboratory of Development and Application of Rural Renewable Energy, Biomass Energy Technology Research Centre, Biogas Institute of Ministry of Agriculture, South Renmin Road, Chengdu, 610041 China; 4 Adaptive Research Farm, Gujranwala, 52250 Pakistan; 5 Department of Botany, University of Education, Lahore, Faisalabad-Campus, Faisalabad, 38000 Pakistan; 6 Tropical Crops Genetic Resources Institute, Chinese Academy of Tropical Agricultural Sciences, Danzhou, 571737 Hainan China

**Keywords:** Drought, *Axonopus compressus*, qRT-PCR, Enrichment analysis, lncRNAs, miRNAs, Transcriptomics

## Abstract

**Background:**

Carpet grass [*Axonopus compressus* (L.)] is an important warm-season perennial grass around the world and is known for its adaptability to varied environmental conditions. However, Carpet grass lacks enough data in public data banks, which confined our comprehension of the mechanisms of environmental adaptations, gene discovery, and development of molecular markers. In current study, the DEGs (differentially expressed genes) in *Axonopus compressus* under drought stress (DS) were identified and compared with CK (control) by RNA-Seq.

**Results:**

A total of 263,835 unigenes were identified in *Axonopus compressus*, and 201,303 (also added to the numbers of the remaining 2 databases) a sequence of unigenes significantly matched in at least one of the seven databases. A total of 153,697 (58.25%) unigenes classified to 144 KEGG pathways, and 7444 unigenes were expressed differentially between DS and CK, of which 4249 were up-regulated and 3195 were down-regulated unigenes. Of the 50 significantly enriched GO terms, 18, 6, and 14 items were related to BP, CC, and MF respectively. Analysis of KEGG enrichment revealed 2569 DEGs involved in 143 different pathways, under drought stress. 2747 DEGs were up-regulated and 2502 DEGs were down-regulated. Moreover, we identified 352 transcription factors (TFs) in *Axonopus compressus*, of which 270 were differentially expressed between CK and DS. The qRT-PCR validation experiment also supports the transcriptional response of *Axonopus compressus* against drought. Accuracy of transcriptome unigenes of *Axonopus compressus* was assessed with BLAST, which showed 3300 sequences of *Axonopus compressus* in the NCBI.

**Conclusion:**

The 7444 unigenes were found to be between DS and CK treatments, which indicate the existence of a strong mechanism of drought tolerance in *Axonopus compressus*. The current findings provide the first framework for further investigations for the particular roles of these unigenes in *Axonopus compressus* in response to drought.

**Supplementary Information:**

The online version contains supplementary material available at 10.1186/s12870-021-02844-7.

## Highlights

1. *Axonopus compressus* can stand severe drought stress by activating the potential defense mechanisms.
2. We investigated the differential transcriptome of drought-stressed and normal *Axonopus compressus* plants
3. New comers involved in the drought-response have been identified
4. The identified drought-responsive genes are not known for other stresses.
5. The identified genes also respond to stress in *Arabidopsis thaliana* in a different manner.

## Background

Axonopus is a genus of the grass family (Paniceae; Poaceae; Triticeae). Approximately 100 species of Axonopus have already been identified [1], which are generally distributed along the shoreline of China, Africa, Alaska, central Asia, eastern Asia, North Sea, South America, and Oceania [2, 3]. About more than 40% (55.5 × 106 km^2^) of the Earth’s surface is occupied by Grasslands world-wide, excluding Greenland and Antarctica [4].

The social and ecological susceptibility to climate change is a giant issue of modern era. The grassland gives an additional opinion about the vulnerability of the ecosystem on account of alterations in policies, socio-economic elements, land use, and local climate [4]. Since the exposure to climatic hazards in various regions has become more apparent, the ecosystems are apt to have a greater degree of vulnerability. Globally, the number of socio-ecological attributes of the eco-systems have changed drastically due to lack of enough soil moisture [5].

Plant growth, nutrient balance, and photosynthetic dynamics have been influenced by drought [6]. Tolerant plant species with drought-responsive mechanisms at various levels including morphological, physiological, and molecular basis can be used to cope with water deficit. Given the important role of carpet-grass in ecosystem protection, scientists (from a macro point of view) are investigating how carpet-grass responds to global changes including drought, salinity, elevated temperature, and CO_2_ elevation [7, 8]. However, little attention has been given to understand the genetic basis of its ecological diversifications, mainly because of the confined genomic resources in carpet-grass. Thus far, only limited ESTs and protein sequences from carpet-grass have been submitted in public online-databases [9]. The discovery of different genes is also lagging, and just a few genes happen to be cloned and functionally authenticated [10, 11].

High-throughput technologies of next-generation sequencing (NGS) like ABI/SOLiD, Roche/454, and Illumina/Solexa have facilitated the production of sizable genome resources at a comparatively low cost [12, 13]. The next-generation sequencing has already been efficiently utilized to create large-scale transcriptome details in a number of plant species like rice [14], Arabidopsis [15, 16], wheat [17], barley [18], and maize [19].

Plants attempt to re-program their metabolic activities and growth while confronting water deficit conditions. It happens to be obvious that plants show distinct and extremely dynamic responses to limited water conditions [20]. These responses varied due to many factors including genotypes, experimental procedures, sampling technique, and time [20, 21]. The molecular mechanisms in response to drought stress have been studied by many scientists to characterize the genes associated with water uptake [22], transporter channels [23, 24], and transcription factors [25]. All these attributes are regarded as the regulators of drought tolerance in plants, although a number of molecular elements and gene networks of drought tolerance mechanisms have been identified but still not fully elucidated [21].

## Methods

### Plant materials and drought treatments

Thirty days old *Axonopus compressus* L. plants with uniform growth were acquired from the germplasm resource library maintained at the Hainan University. The germplasm resource library is maintained by Key Laboratory of Genetics and Germplasm Innovation of Tropical Special Forest Trees and Ornamental Plants, Ministry of Education, College of Forestry, Hainan University, Haikou 570,228, P. R. China. Plants were grown in chambers with controlled conditions (27 °C, 16-h day length, and 60% RH). A total of 90 *Axonopus compressus* L. plants were primarily divided into two groups as CK (control group) and DS (drought experiment group). There were 5 cuttings in each pot and 20 cuttings in one replicate group. This experiment included 3 repeats named R1, R2, and R3. The drought treatments were induced by using the Polyethylene-glycol (PEG-8000). The PEG-8000 solution was replaced after 48 h. At eight time points (0 h, 6 h, 12 h, 18 h, 24 h, 36 h, 48 h, and 72 h after induction of drought treatment) the functional leaves (3rd to 5th mature leaf) were chosen randomly from the plants of CK and DS for physiological attributes. For transcriptome sequencing, samples were instantly frozen in liquid nitrogen, stored at − 80 °C, and finally sent to Metware Biotechnology Co., Ltd., Gaoxin Road, East Lake High-tech Zone, Wuhan, China.

### Measurement of physiological traits

The physiological traits were recorded in different time intervals of 0 h, 6 h, 12 h, 18 h, 24 h, 36 h, 48 h, and 72 h after induction of drought. The leaf water potential (LWP) was measured by Scholander chamber (SF-PRES-70, Solfranc Tecnologías SL, Vila-Seca, Spain). Electrolyte leakage (EL) was measured by estimating the electrical conductivity [26]. In the solution, the electrolyte leakage (S1) was measured using a conducti-meter after 22 h of floating at room temperature (Mettler-Toledo Instruments Co., Ltd., Shanghai, China). Total conductivity (S2) was collected after the flasks were placed in a boiling water bath for 30 min. Relative water content (RWC) was also estimated, as described in Liu et al. [27]. For each plant, three independent replicates were collected for LWP, EL, and RWC. All measurements were recorded at three biological replicates.

### Extraction of RNA of *Axonopus compressus*, library preparation

Total RNA of *Axonopus compressus* was extracted by employing the Trizol method (Invitrogen, Carlsbad, CA, USA). The RNA integrity, purity and concentration were accessed by the Nanodrop spectrophotometer (Qubit 2.0, Agilent 2100). The sequencing libraries of *Axonopus compressus* were created by NEBNext®Ultra™ RNA Library Prep Kit for Illumina® (New England Biolabs, Ipswich, MA, USA), as suggested by the manufacturer. The first strand of cDNA was synthesized by random hexamer primer, afterwards, the dNTPs, buffer, DNA polymerase I, and RNase H were used to generate cDNA’s second strand. Finally, the AMPure XP beads were used to purify cDNAs and afterward the end-repair and single nucleotide A (adenine) addition, the cDNA libraries (qualified) were developed by PCR technique. The Qubit 2.0 was used for primary quantitation, followed by Agilent 2100, which was employed to identify libraries insert size. Thereafter, the q-PCR technique had been used for accurate quantification of the effective strength of the libraries (effective library concentration > 2 nM) to guarantee the library quality. After passing through a series of screening steps, the high-throughput sequencing was executed by Illumina Hiseq Xten. The protein-coding region accuracy and completeness assessment in *Axonopus compressus* was performed by using BUSCO analysis (BUSCO_v2/v3) [28].

### Validation of RNA-seq data by qRT-PCR

The RNA was extracted from the CK and DS groups and used to develop the cDNA library. First-strand synthesis was carried out via MonScript (Monad) according to the suggested protocol by the manufacturer. Biosystems-7500 (Thermofisher Scientific) was used to conduct the qRT-PCR and the primer pairs are listed in Additional file 1; Table S1. The gene expression was quantified through CT-method, and the Actin1 was used as a reference for normalization.

## Results

### Effect of drought stress on physiological indicators in *Axonopus compressus*

**Fig. 1:**
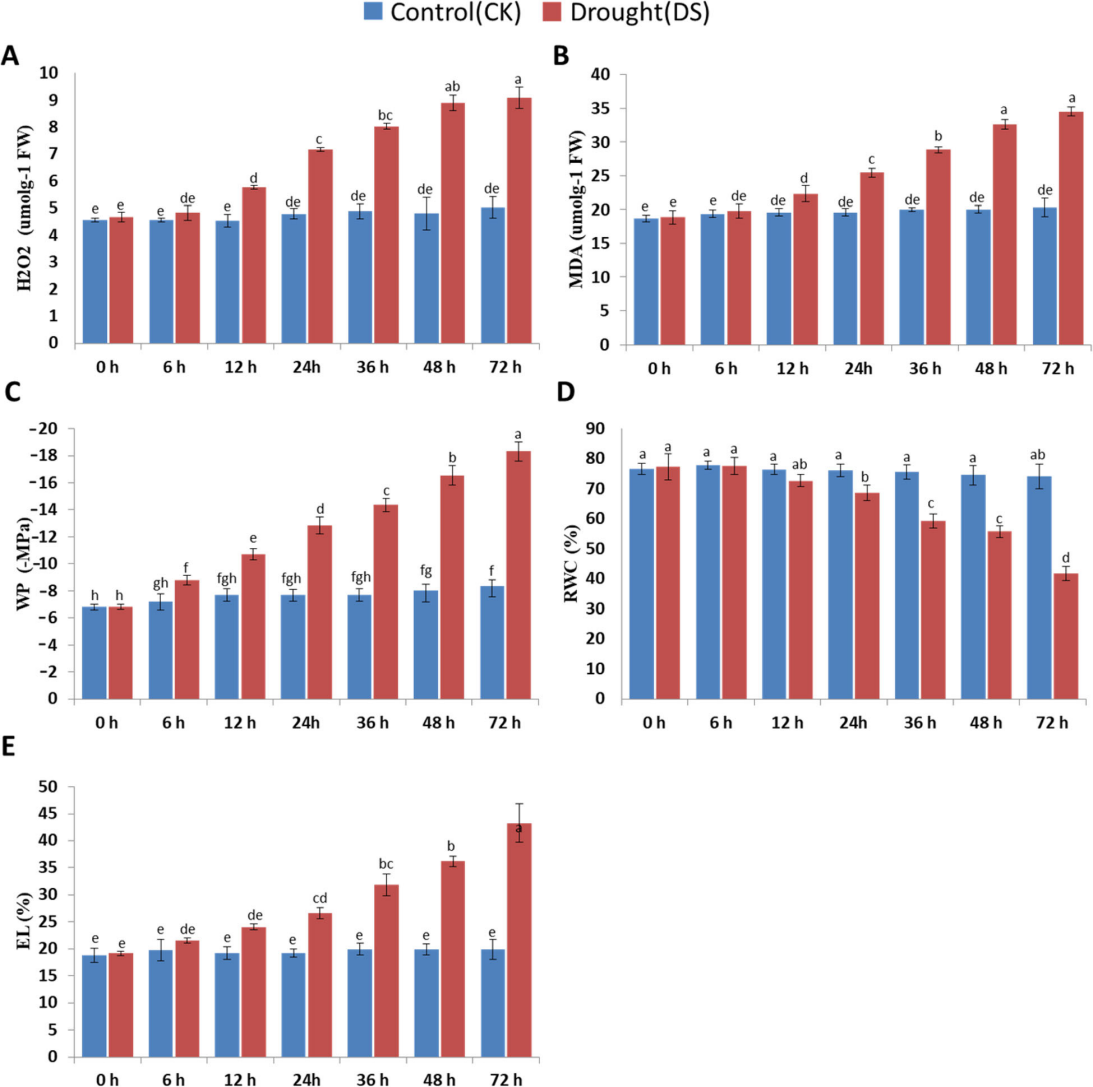
Effect of drought on the physiological traits of the *Axonopus compressus.* A; effect of drought on H_2_O_2_ production, B; Effect of drought stress on MDA content, C; drought-induced alteration in WP, D; Effect of drought on leaf RWC, E; drought stress effects of the electrolyte leakage (EL) in *Axonopus compressus*

In Fig. 1, it was revealed that the rate of H_2_O_2_ (Fig. 1a) production and MDA concentration (Fig. 1b) increased in drought treated plants. The MDA values and H_2_O_2_ production rate increase with the prolonged drought stress treatment, whereas MDA values and the production rate of H_2_O_2_ at 36 h after drought induction were significantly increased (*p* < 0.01). Similarly, the leaf water potential and relative water content decrease significantly under drought, contrarily, the electrolyte leakage increased over the drought period in *Axonopus compressus*. As in the case of MDA and H_2_O_2_, the values of EL were also increased as the time of drought stress increase. Whilst, the values of leaf water potential and relative water content decrease with the prolonged drought stress (Fig. 1c).

### RNA-Seq of *Axonopus compressus* and de novo assembly

RNA-Seq of the six libraries of cDNA (DS and CK with three replicates each) resulted in 352.84 million raw reads, of which approximately 347.73 million clean reads de-novo compiled into contigs utilizing Trinity software, tend to range from 54.90 million to 62.25 million reads for each library and approximately 97% of reads with a quality score of Q20 (99% accuracy) (Additional file 1; Table S2). The contigs were assembled into 278,042 unigenes with an average length of 1066 bp and an N50 length of 1430 bp. All unigenes were over 200 bp long, of which 11.77% (32,718 unigenes) were 2000 bp long. We evaluated the assembled data of transcriptome by employing Benchmarking Universal Single-Copy Orthologs (BUSCO) package. The number of total BUSCOs searched was 1440. The number of complete BUSCOs in the combined transcriptome was 1080 (75.00%), and only 198 core genes (13.75%) are fragmented in *Axonopus compressus* (Additional file 2; Fig. S1).

### Functional annotation and classification of unigenes

**Fig. 2:**
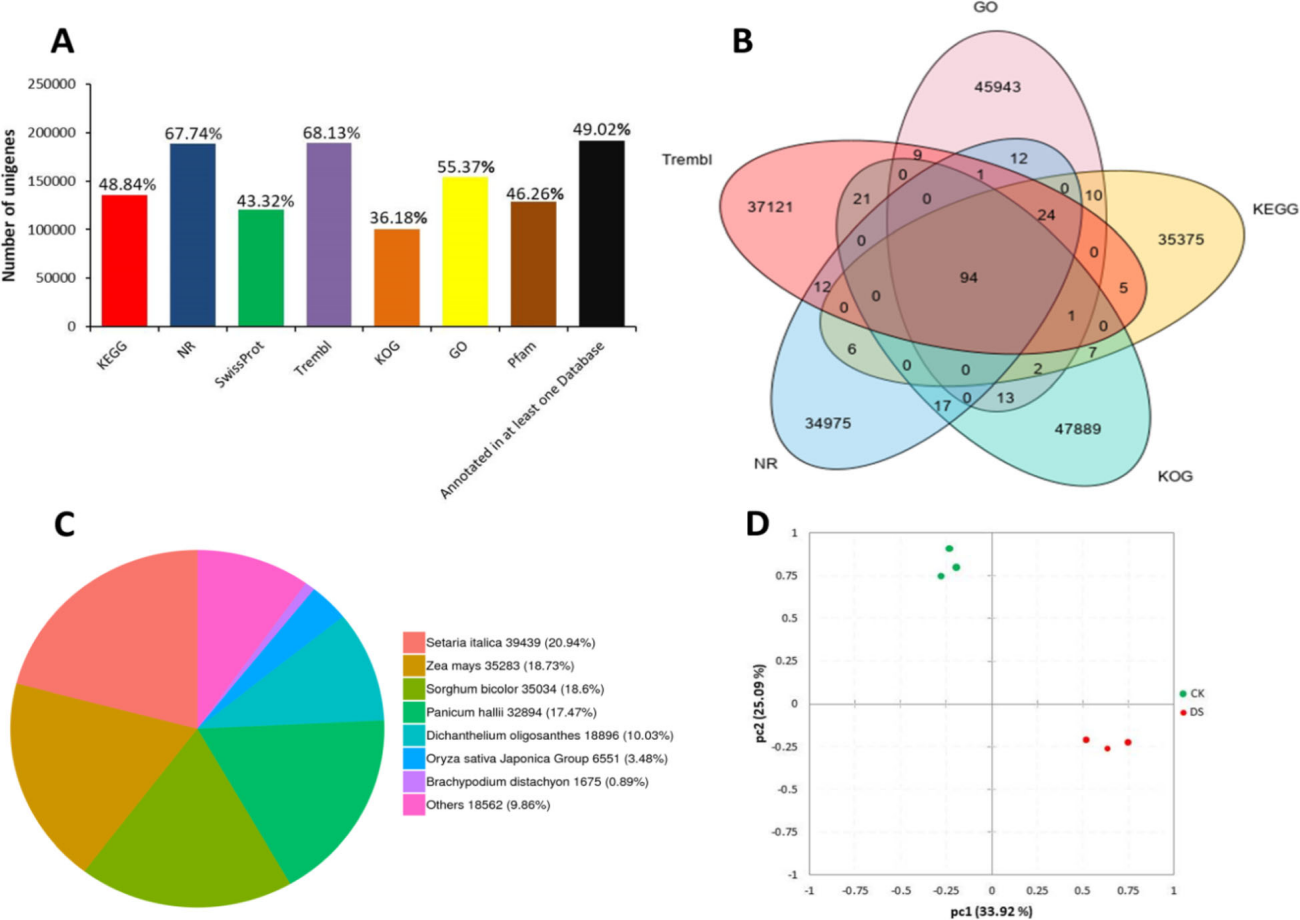
**a;** Unigenes matched in seven databases, **b;** Venn diagram of differential BLAST results for *Axonopus compressus*. The number of unigenes annotated with five databases and the co-annotated gene number are also presented, **c;** Homologous species distribution of *Axonopus compressus* annotated in the NR database, **d;** Principal component analysis of transcriptome data

The assembly of unigenes from *Axonopus compressus* was annotated by BLASTX (E-value < 10^− 5^) in different databases. A total of 135,716 (48.81%), 188,334 (67.64%), 120,455 (43.32%), 189,422 (68.13%), 100,588 (36.18%), 153,958 (55.37%) and 128,626 (46.26%) unigenes would have significant levels (E-value < 10^− 5^) against KEGG, NR, Swissprot, Trembl, KOG, GO, and Pfam, respectively (Fig. 2a). Out of 278,042 unique of high-quality sequences, 191,893 (69.03%) unigenes complemented a sequence significantly in at least one of the seven public databases, The five key online databases (GO, KEGG, KOG, NR, and Trembl) were picked out of seven databases to draw a Venn diagram (Fig. 2b), the unigenes with significant values (E-value10^− 5^) are also noticed at each junction of the Venn diagram, in which 94 unigenes corresponds to five databases.

The alignment of sequence homology revealed that 39,439 (20.94%) sequences that were found in resemblance with *Setaria italica*; 35,283 (18.73%) sequences had significant hits for *Zea mays*, followed by *Sorghum bicolor* (35,034, 18.6%), *Penicum hallii* (23,894, 17.47%), *Dichanthelium oligosanthes* (18,896, 10.03%) and *Oryza sativa japonica* group (6551, 3.48%). *Brachypodium dischyon* which included 1675 (0.89) and 9.86% of the sequences (18562) were homologous to other species (Fig. 2c). The expression of genes has biological variability between different individuals, and there are differences in the degree of expression between genes. The reads per kilobase of exon model per million of aligned reads (FPKM) values were calculated as normalized expression estimates for each gene model in each sample. To evaluate the major spectral variance between CK and DS treatment samples, we performed PCA (Fig. 2d). Results showed a distinct separation of CK to DS sample treatments, which reveals that both groups of the treatment samples exhibit different spectral positions on the PCA chart (Fig. 2d).

**Fig. 3:**
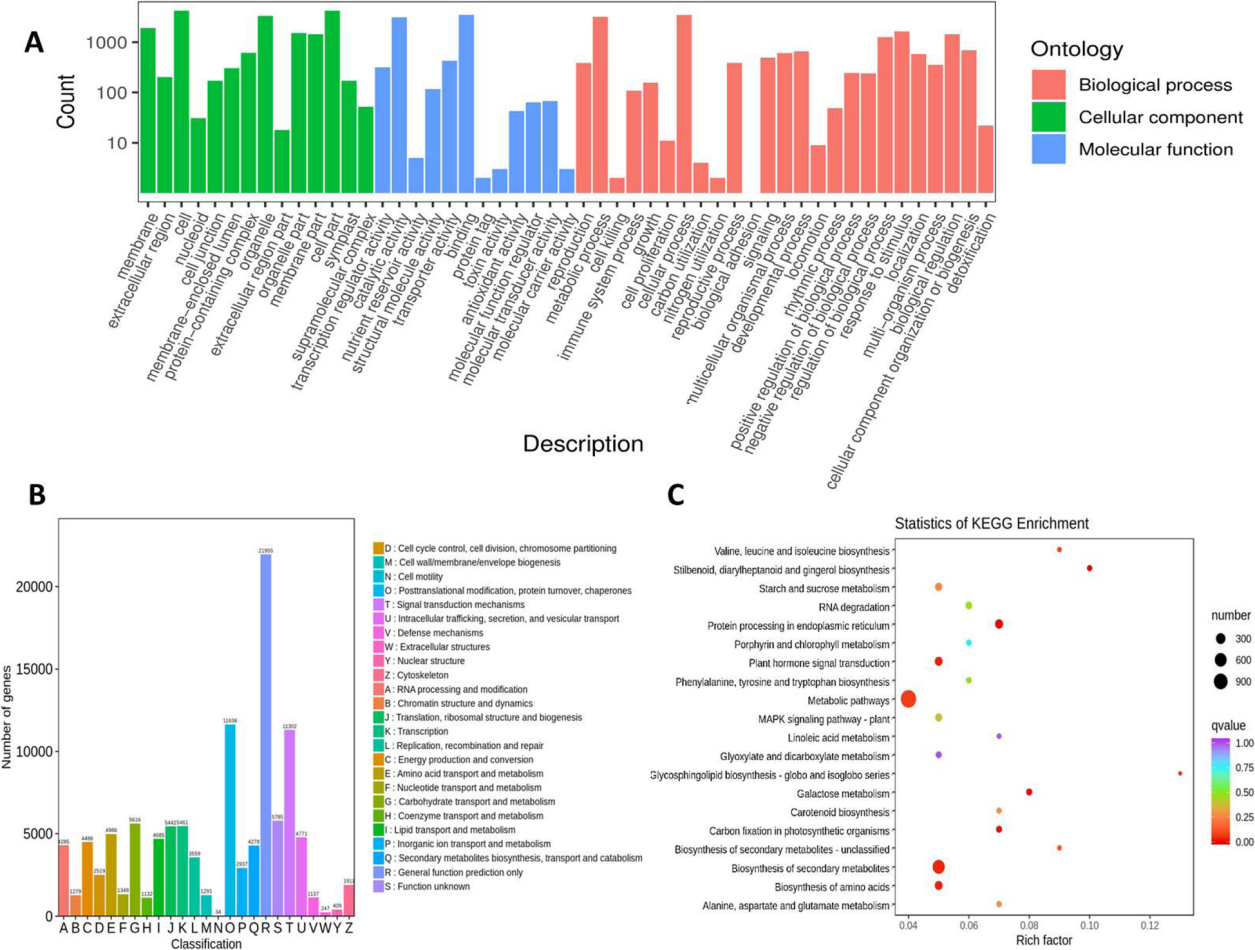
**a;** Functional classification of GO terms for *Axonopus compressus*. The number of genes in a specific sub-category within the main category is shown on the y-axis; the name of the sub-category is shown on the x-axis, **b;** KOG functional classification of the *Axonopus compressus* transcriptome. The unigenes with significant homologies in the KOG database (E-value < 10^− 5^) were classified into 26 KOG categories. Capital letters on the x-axis indicate KOG categories on the right side of the histogram **c;** KEGG pathway enrichment of DEGs in *Axonopus compressus*. The graph shows only the top 20 enriched pathways comparing DS with CK; different colors denote different Q-Values, and the size of the bubble represents the number of DEGs

To demonstrate the accuracy of the unigenes prediction, the nucleotide sequences from the transcriptome of *Axonopus compressus* were used as a query in a BLAST search with a threshold E-value < 10^− 5^. The results showed 3300 sequences of *Axonopus compressus* in the NCBI database. This analysis renders an assessment for the consistency of unigenes sequences in the current dataset. Based on the high-score BLASTx matches in the GO proteins database, The BLASTx high-score Predicated matches in the computer database of Go proteins were confirmed, and a total of 153,958 unigenes were categorized with Blast2GO (E-value < 10^− 5^) and were designated at least once in GO. As shown in Fig. 3, the unigenes referred to three main categories of GO and 59 subcategories, namely biological processes (BP), with 28 main sub-classes (405,025 unigenes); cellular compartments (CC), with 18 main sub-classes (460,094 unigenes); and molecular functions (MF), with 13 main sub-classes (194,989 unigenes).

Cellular processes (21.43%) were the biggest subgroups in the category of biological processes, metabolic process (19.70%), biological regulation (9.24%), response to stimulus (9.22%), and regulation of biological process (8.36%). The largest subgroups in the cellular component category were cell (22.53%) followed by cell part (22.47%), organelle (17.22%), and membranes (11.72%) respectively. Similarly, the main subgroups in the molecular function category were binding and catalytic activity, which contribute 77.78 and 69.28% respectively, and 115,199 unigenes associated with molecular function. Within the *Axonopus compressus* unigenes, 112,492 (42.64%) were classified (E-value *<* 10–5) into twenty-six KOG clusters (Fig. 3b). The biggest groups which includes; 1) general function prediction, only (21,955 genes, 19.52%); 2) posttranslational modification, protein turnover, and chaperones (11,636 genes, 10.34%); 3) signal transduction mechanisms (11,302 genes, 10.05%); 4) Function unknown (5785 genes, 5.14%); and 5) carbohydrate transport and metabolism (5616 genes, 4.99%).

### Metabolic pathway analysis of *Axonopus compressus* by KEGG

A total of 135,717 (51.44%) of the 263,835 *Axonopus compressus* unigenes possessed significant correspondence in KO. All these unigenes have been restricted to 103 KEGG pathways in 5 major categories (Fig. 3b). The pathways of KEGG (which includes 4303 unigenes) were the members of a major group, metabolism (D), 921 associated to genetic information processing (C), 184 related to cellular processes (A), 347 involved to environmental information (B), and 151 related to category (E) of organism systems (Additional file 2; Fig. S2).

### CDS prediction in *Axonopus compressus*

The BLASTx protein database (NR and SwissProt database) identified 278,042 unigenous CDSs, of which 23,652 unigenes were larger than 500 bp, 12,520 unigenes were larger than 1000 bp, and 32,718 unigenes were larger than 2000 bp. In addition, 43,713 unigenes were not linked to the NR and SwissProt database systems. The Estscan (Version; 3.0.3) software was used to interpret their ORF, frequency distribution, length, and related amino acid sequences of the unigene CDSs.

### Differentially expressed genes (DEGs) analysis of *Axonopus compressus*

**Fig. 4:**
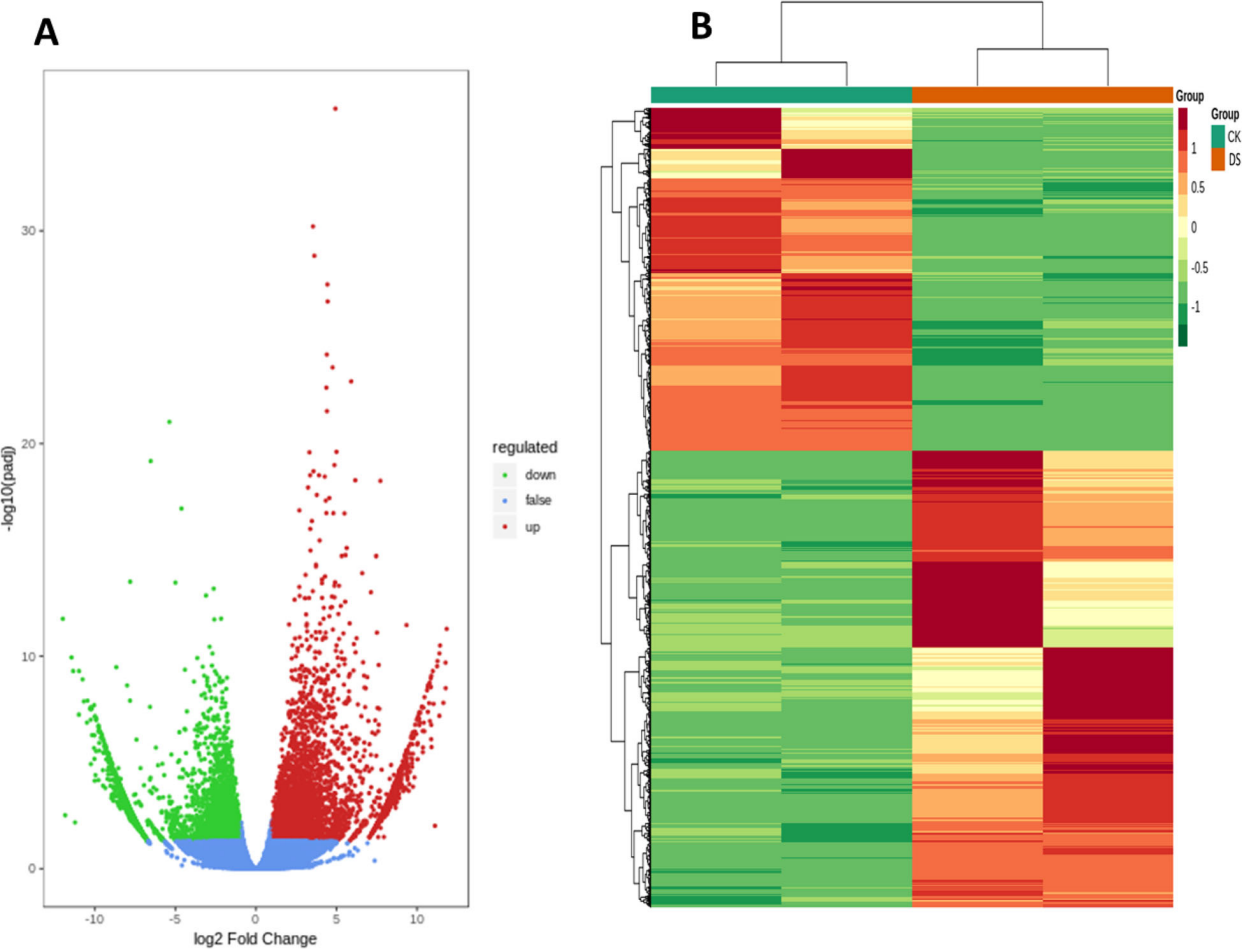
**a** Volcano plot of differentially expressed unigenes of *Axonopus compressus*. The numbers of up and down-regulated unigenes in CK and DS are shown. Red indicates up-regulated genes, green represents down-regulated unigenes, and blue represents no significant difference in gene expression, **b;** cluster analysis of differentially expressed unigenes of *Axonopus compressus* in responses to CK and DS treatments

Amongst the differentially expressed unigenes, the expression of 7444 differs substantially between samples treated with drought-stress (DS) and control (CK) samples. Under drought treatment, 4249 numbers were up-regulated and 3195 numbers were down-regulated (*p <* 0.05) (Fig. 4a). The expression profiles of DEGs were also presented through cluster analysis that showed significantly different responses of CK and DS treatments in the *Axonopus compressus* (Fig. 4b).

### GO pathway enrichment analysis

By employing the Gene Ontology (GO) and the DEG enrichment analysis in *Axonopus compressus*, 23,766 DEGs were categorized into three GO groups and 4592 numbers associated the DS with CK (number of DEGs annotated in more than one term), out of which 2766 items that were associated to BP, 477 were linked with CC and 1349 were related to MF.

The key 50 DEGs dramatically enriched in three GO categories are presented in Additional file 2; Fig. S4. In the top 50 significantly enriched identities (Corrected *P*-Value*<* 0.05), 18 were found to be linked with BP [carotenoid biosynthetic process GO:0016117, carotenoid metabolic process GO:0016116, cellular amine metabolic process GO:0044106, cellular biogenic amine metabolic process GO:0006576, cellular response to heat GO:0034605, cellular transition metal ion homeostasis GO:0046916, glutamine family amino acid biosynthetic process GO:0009084, Group II intron splicing GO:0000373, heat acclimation GO:0010286, oligosaccharide catabolic process GO:0009313, raffinose catabolic process GO:0034484, raffinose metabolic process GO:0033530, regulation of seed germination GO:0010029, regulation of seedling development GO:1900140, response to high light intensity GO:0009644, response to hydrogen peroxide GO:0042542, tetraterpenoid biosynthetic process GO:0016109, tetraterpenoid metabolic process GO:0016108], and 6 items were related to CC [chloroplast nucleoid GO:0042644, DNA packaging complex GO:0044815, Nucleoid GO:0009295, Nucleosome GO:0000786, plastid nucleoid GO:0042646, protein-DNA complex GO:0032993], and 14 items were related to MF [4-coumarate-CoA ligase activity GO:0016207, alpha-galactosidase activity GO:0004557, arogenate dehydratase activity GO:0047769, carbon-nitrogen ligase activity, with glutamine as amido-N-donor GO:0016884, ferric iron binding GO:0008199, ferroxidase activity GO:0004322, galactosidase activity GO:0015925, oxidoreductase activity, acting on single donors with incorporation of molecular oxygen GO:0016701, oxidoreductase response, acting on single donors with molecular oxygen incorporation, incorporation of two atoms of oxygen GO:0016702, oxidoreductase response, oxidizing metal ions, oxygen as acceptor GO:0016724, raffinose alpha-galactosidase activity GO:0052692, water channel activity GO:0015250, water transmembrane transporter activity GO:0005372] (Additional file 3).

### *Axonopus compressus* KEGG pathway enrichment analysis

The KEGG pathway enrichment analysis for DEGs revealed that 2569 DEGs participated in 143 various types of pathways in *Axonopus compressus.* By comparing DS with CK, 2747 DEGs were found to be up-regulated and 2502 DEGs have been identified as down-regulated in deficit water. The twenty key pathways which were found significantly enriched by comparing DS with CK are displayed in (Fig. 3c). The pathways includes Valine, leucine and isoleucine biosynthesis (215 unigenes), Stilbenoid, diarylheptanoid and gingerol biosynthesis (330 unigenes), Starch and sucrose metabolism (1642 unigenes), RNA degradation (1536 unigenes), Protein processing in endoplasmic reticulum (1628 unigenes), Porphyrin and chlorophyll metabolism (559 unigenes), Plant hormone signal transduction (1672 unigenes), Phenylalanine, tyrosine and tryptophan biosynthesis (601 unigenes), Metabolic pathways (1659 unigenes), MAPK signaling pathway plant (1661 unigenes), Linoleic acid metabolism (323 unigenes), Glyoxylate and dicarboxylate metabolism (1050 unigenes), Glycosphingolipid biosynthesis - globo and isoglobo series (103 unigenes), Galactose metabolism (735 unigenes), Carotenoid biosynthesis (506 unigenes), Carbon fixation in photosynthetic organisms (820 unigenes), Biosynthesis of secondary metabolites - unclassified (223 unigenes), Biosynthesis of secondary metabolites (1661 unigenes), Biosynthesis of amino acids (1655 unigenes), Alanine, aspartate and glutamate metabolism (639 unigenes) (Additional file 2; Fig. S3).

## Drought stress associated differentially expressed transcription factors (TFs)

The transcription factors play a crucial role in the growth and development of plants and therefore can stimulate and/or suppress transcriptional gene expression to sustain normal physiological functions in deficit water stress. In the current study, 352 TFs were observed in *Axonopus compressus* based on iTAK, including 270 transcription factors and 81 transcription regulatory factors that were associated with 32 and 16 families, respectively (Additional file 1; Table S3, S4). The differential analysis revealed that 270 transcription factors were different as shown in the comparison between DS and CK, 216 transcription factors have been noted to be up-regulated while 54 transcription factors were found down-regulated.

### qRT-PCR validation of transcripts in *Axonopus compressus* under drought

**Fig. 5:**
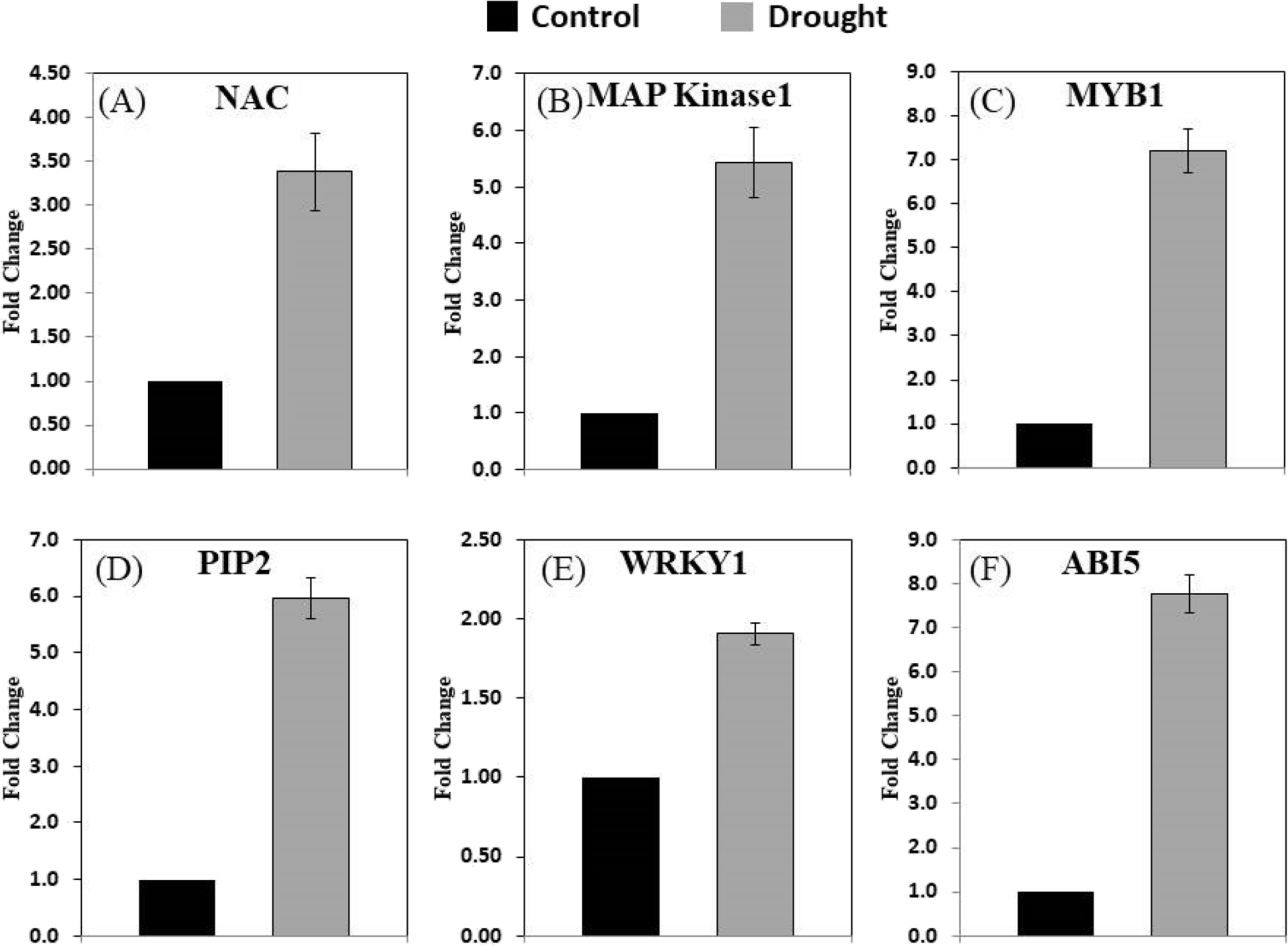
The qRT-PCR expression validation of drought-responsive genes in *A. compressus*. The bars showed relative expression of **a;** NAC, **b;** MAP Kinase1, **c;** MYB1, **d;** PIP2, **e;** WRKY1, **f;** ABI5

To check the validity of RNA-seq results, the expression of six transcripts for the three biological replicates was analyzed by qRT-PCR (Fig. 5). All the genes were identified as significantly different expression values (DEGs). The findings of qRT-PCR revealed that the pattern of expression of these genes was identical with the findings in the RNA-seq analysis. To validate the results, the six drought-responsive genes (NAC, MAP Kinase1, MYB2, PIP1, WRKY1, ABI5) were verified using qRT-PCR. The different genes were amplified to compare the gene expression and RNA-seq results, and the findings of this study revealed that the expression of drought-responsive genes supporting the results of the RNA-seq.

## Discussion

Plants have a sessile lifestyle, and usually confront adverse environmental circumstances such as drought, cold, heat, salt, and floods. Deficit water is the most notable environmental determinant influencing crop production around the globe [29]. To improve the potential tolerance mechanism against deficit water stress and also to develop drought-resistance in plants, many experts have attempted to elucidate the mechanisms of stress signaling in plants conferring several perspectives [30, 31]. Plant defense mechanisms for abiotic stress are already being studied, but the response to drought has always been a complex event with several critical indicators to be examined.

Drought stress significantly increases ROS formation and imposes oxidative stress on the plant [26, 32]. Since lipid peroxidation is one of the earliest indicators of oxidative damage, MDA was estimated as an index for the production of drought-induced ROS [32]. A significant rise in MDA values and H_2_O_2_ concentration were noticed in *Axonopus compressus* leaves in drought conditions (Fig. 1b). Electrolyte leakage increased significantly with a decline in water potential (Fig. 1e), which proposed that plant systems had been damaged by drought, as studied previously [33]. Drought might have had an impact on the physical membranous structure of the lipid bilayer by inducing phase destruction [34].

To sustain under severe environmental conditions, plants have developed an obscure regularity mechanism through a series of evolutionary advancements at varied levels to respond to external signals and prompt transduction of stress signals, directing to a set of responses at different levels, i.e., physio- morphological, biochemical, and omic levels [35, 36]. As a strong drought-responsive plant, *Axonopus compressus* developed a series of morphological features to acclimate the deficit water conditions. Drought stress can drive the plant to change the metabolic status of cutin and wax, which is ultimately accomplished through the associated gene-expression regulation [37] In current study, the unigene responsible for the wax biosynthesis (Cluster-37,496.124448) was enriched under DS in comparison with CK and also up-regulated at the transcript level. As a distinctive morphological feature of protection against drought stress, cutin and wax play a critical role during water deficit conditions by restricting loss of water by means of leaf epidermis (non-stomatal transpiration) [38, 39], thereby enhancing water use capability in plants. In our study, the transcription factors that belong to the family AP2/ERF-ERF, MYB-related, zinc-finger, NF-X1 were found to be up-regulated. These transcription factors were known to regulate the biosynthesis of plant cutin and wax [40].

In water deficit conditions, plants receive stress signals first, and thereafter, several pathways in a sequence are triggered by phytohormones [41]. Subsequently, plant hormones play an important role in responding to scarce water conditions, and amongst the phytohormones, ABA is known to be a major homeostatic controller of abiotic stresses. In water deficit conditions, plants increase ABA content and close stomata to avoid the excessive water loss by transpiration across stomata [42], such a high level of water loss would sometimes continue to occur even after transient recovery of leaf water status [43].

In current study, we observed 8 unigenes, which encode NCED4 (9-cis-epoxycarotenoid dioxygenase) (Cluster-37,496.127826), a crucial enzyme accountable for ABA biosynthesis (GO:0009688). Amongst 8 unigenes, 4 were up-regulated and exhibited substantially different expression levels between DS and CK. Such comportment in response to drought stress indicates that *Axonopus compressus* plants had perceived the signals to produce ABA and to translocate it to the above-ground parts. The transportation mechanism in the stem comes into action and triggers short-term responses such as stomatal closure [41, 44], which potentially help *Axonopus compressus* to avert dehydration and improve water holding capacity under drought stress. Accordingly, the understanding of stress signaling and subsequent molecular mechanisms in *Axonopus compressus* need to be elucidated in succeeding experiments to reveal the drought-resistance mechanism.

When plants respond to drought, the rise in amounts of ABA leads to binding with PYR/PYL, which perform a significant role in quantitative regulation of stomatal movements and transcriptional response to ABA [45, 46]. The PYR/PYL reconstructs the PYR/PYL protein conformations, and this alteration enables the PYR/PYL to interplay with 2 C protein phosphatase (PP2C), negative regulator type, to construct a substitute complex (ABA-PYR/PYL-PP2C). The ABA-PYR/PYL-PP2C- complex may impede PP2C activity and trigger SNF1-related protein kinase 2 (SnRK2s), a positive regulator. While the PP2C also inhibits the activity of SnRK2s [47]. Such mechanism stimulates the stress-responsive genes to down-regulate its expression and supports the plants to acclimate water deficit conditions [48]. 715 PP2C were found in our experiments, from which 82 genes were remarkably down-regulated, and 17 SnRK2 were also identified. This symbolizes that under water deficit, the low expression of PP2C in *Axonopus compressus* rescue the suppression of SnRK, and its expression promotes the closing of stomata. The SnRK expression also triggers the stress-related genes towards downstream and support *Axonopus compressus* keeps normal growth and persistence in drought.

In plants, the stress responding genes can easily be divided into two groups i.e., functional and regulatory [49]: The functional proteins are related to small molecular osmolytes (i.e., proline, betaine, and soluble sugar), enzyme protectants (POD, SOD and CAT), late embryogenesis abundant (LEA), and aquaporins. The regulatory proteins include TFs, phosphatases, protein kinases, and phospholipid metabolic enzymes that can regulate the stress-related expression of genes under abiotic stresses. It indicates that during water deficit conditions, the lowest expression of PP2C in *Axonopus compressus* would rescue the SnRK suppression. The PP2C expression would induce closing of stomata and trigger the downstream drought stress-related genes and assist the *Axonopus compressus* to sustain unhampered growth under drought. These proteins will prevent the plant cells from damaging encounters of stress by managing turgor pressure, including oxygen-free radicals scavenging and the structural intracellular bio-macromolecules protection [50, 51].

It has been confirmed that members of the TF families i.e., MYB, AP2, DREB, bHLH, PLATZ, bZIP, C2H2, NAC WRKY, and HB are involved in plant stress-response mechanisms [52]. In our research, we found 270 DEGs in *Axonopus compressus* TFs, among them, 216 were up-regulated and 54 were down-regulated. It is obvious from the results that large numbers of TF expressions differed significantly under water deficit.

**Fig. 6:**
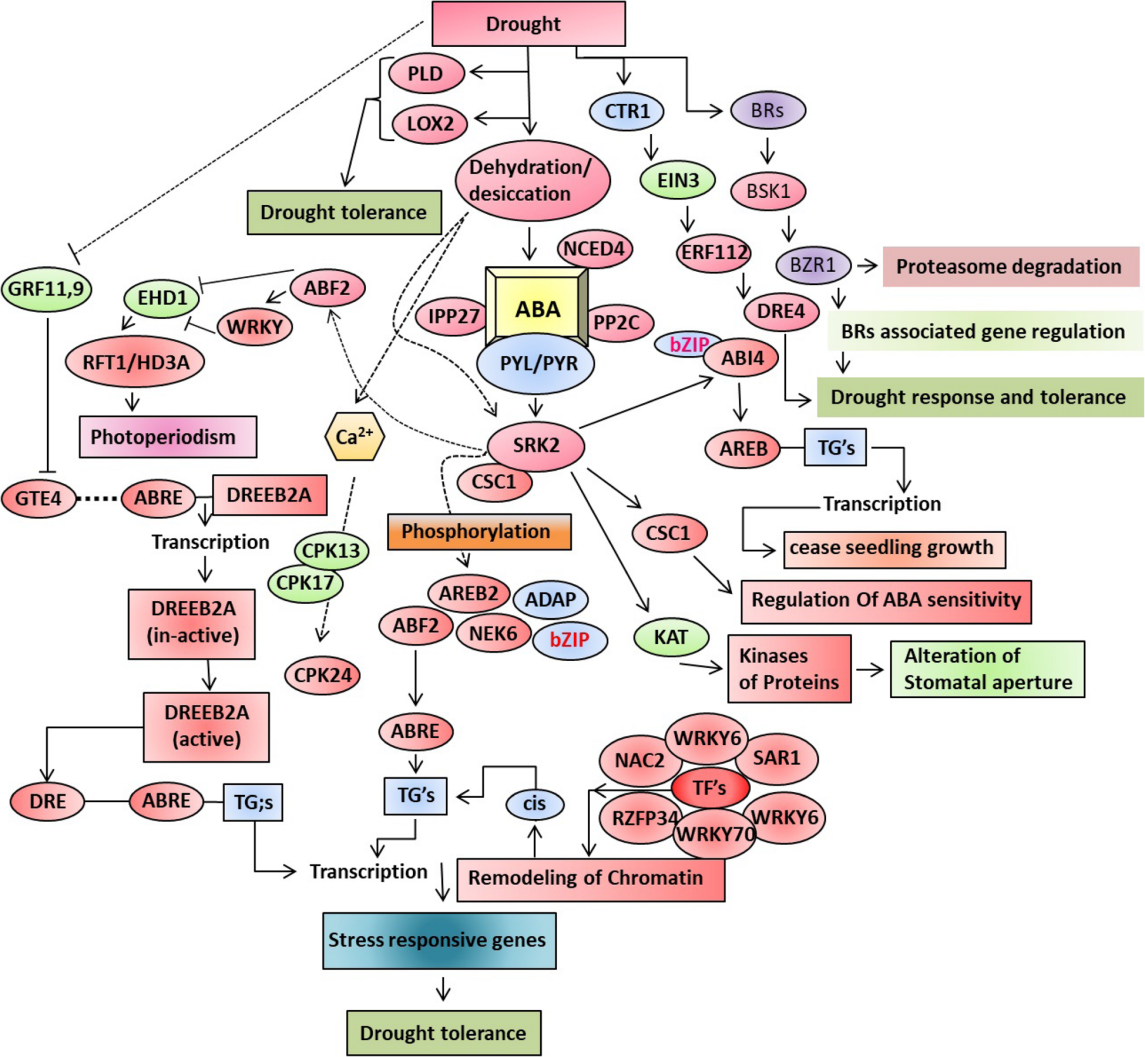
Schematic diagram of potential defense mechanisms for drought stress response and tolerance pathways in carpet-grass (*Axonopus compressus*)

In the DEGs *HARBI1, EEF1A, SUMO, ATXR3, SEY1, NLR, COMM, DHAR3, GUF1, SFH5, SHOC2, MAPK’s, FYVE, PIP, LSM14, PERQ, BRCA1, TIM22, STY46-like, SPN1, NEDD8, DDX21, Meis2, RAD50, MAG2, MON1,* and *AKT2* are all down-regulated. The *RXW8, DHAR2-like, EFR3, MOCS2A, GDSL, ANP1-like, TBC1, ERCC-6, ZDHHC2, EXO84B, BAHD, SHOC2, XBAT32, RNF38, SPIRAL1, NDRG1, HESO1, NBR1, STY46, TNNI3K, PP2C26, HVA22* and *DAD1* are all up-regulated. The TF families i.e., GATA, ATF/CREB, ABI, bZIP, WRKY, EREBP, AP2-like, PTI6, TRAF, ARF1, ATF/CREB, ABA-responsive, RING, MYB, NAC, TUB, HSF, GRAS, C2H2, bZIP, SET, AP2/ERF-ERF, AUX/IAA, SNF2, and PTI6 some of them were up-regulated and some of them were down-regulated. A schematic diagram of potential defense mechanism and drought tolerance involving different pathways in *Axonopus compressus* is shown in Fig. 6. Several unigenes (belonging to a similar family) were reported to have distinct expression models under water deficit conditions. It might symbolize various characteristic functions in response to drought [47, 53]. A particular transcription factor can be associated with one and/or more than one category and even other groups [54]. It reveals the complexity by which transcriptional categories of genes control the drought responses of *Axonopus compressus*. The current study provides helpful data for additional functional characterization of the TFs to develop tolerance in this plant.

During signal transduction in plants under stress conditions, phosphorylation and de-phosphorylation are very important mechanisms for post-translational protein modifications [55]. Phosphorylation and dephosphorylation can trigger a number of proteins and enzymes with regulatory functions to control a wide range of cellular mechanisms or signaling process. It has been established that protein phosphorylation plays a significant role in drought response to halt or initiate the enzyme activity, for adjusting the concentration of resultant proteins or intracellular enzyme activity [55]. It was reported that Arabidopsis has numrous kinases [56]. From which the CDPK (calcium-dependent protein kinase) and MAPK (Mitogen-activated protein kinase) are considered critical signaling mechanisms in plants under stress. The stress signals were transduced into cellular processes by MAPK, succeeding phosphorylation processes of distinct downstream proteins for turning on and/or off their activities [56, 57]. In our study, 11,089 protein kinases were observed, of which 344 were DEGs (182 up-regulated and 162 down-regulated). There were 16 DEGs encoding MAPKs (9 up regulated and 7 down regulated) and 8 DEGs for CDPKs (2 up regulated and 6 down regulated). It suggests that the MAPKs and CDPKs pathways were engaged in the signaling of drought stress response of *Axonopus compressus*. When plants face water deficit conditions, osmo-tension would provoke ROS built-up in cells. Plants evolved the defense mechanism to extinguish the ROS and alleviate cellular damage. This defense mechanism comprises of SOD, POD, and CAT. The current study indentified DEGs related to 5 SODs, 3 CATs and 25 PODs. In addition, proteins of small-molecules such as proline, betaine, and LEA (late embryogenesis abundant) likewise play significant roles in restraining the cells from the damaging effects of drought. It was onserved that 370 unigenes can encode LEA. We observed 19 DEGs related to LEAs in *Axonopus compressus* all of which were up-regulated under stress. Therefore, the unigenes are crucial for developing sustainability against the water shortage for *Axonopus compressus.* Additionally, the osmotic regulation ability of plants to hamper water loss is an additional fundamental feature to survive in drought. The bidirectional water channel AQP (aquaporin) in plants is responsible for trans-membrane water mobility and long-distance water transportation. It is established that plants have the potential to defy the numerous stress circumstances through regulating the AQP proteins [58]. In *Axonopus compressus*, we observed 363 unigenes coding AQPs, including 31 differentially expressed unigenes. Majority of AQP’s showed a reduced expression, which indicates a potential decline in AQP’s activity. The decrease in AQPs expression is expected to correlate with limited moisture loss. It may sustain water-related homeostatic processes. Consequently, improving tolerance against drought in *Axonopus compressus*.

## Conclusion

Under drought conditions, processing of stress signals, signal transduction, regulating gene expression and the subsequent downstream functional genes are essential variables in plant response to adversity. In current study, we observed 263,835 unigenes in *Axonopus compressus* based on RNA-Seq, in which 7445 were differentially expressed unigenes (DS and CK). Overall, 2747 were up-regulated and 2502 down-regulated unigenes. In addition, 352 (TFs) were found to be differentially expressed in *Axonopus compressus* (DS and CK). Current findings indicate that these genes are involved in the campaign of resistance to drought stress and have a highly significant and complicated role. This study provides valuable data for the molecular mechanisms underlying drought tolerance. The qRT-PCR validation of transcripts sets the basis for further investigations of the gene regulatory networks under drought stress and other abiotic stress factors in *Axonopus compressus*.

## Supplementary Information

**Additional file 1.**

**Additional file 2.**

**Additional file 3.**

## Data Availability

The datasets generated and/or analysed during the current study are included in this article, its supplementary information files and in the [NCBI] repository with Accession: PRJNA688376 [https://www.ncbi.nlm.nih.gov/sra/PRJNA688376].

## Abbreviations

DEGs: Differentially expressed genes
DEPs: Differentially expressed proteins
FPKM: Fragments Per Kilobase Million
GO: Gene Ontology
KO: KEGG Orthology
GST: Glutathione S-transferase
DS: Drought Stress
CK: Control
NGS: Next Generation Sequencing
KEGG: Kyoto Encyclopedia of Genes and Genomes
MAPK: Mitogen-activated protein kinase
NDEGs: No significant difference in genes.
RNA-seq: RNA sequencing.
ROS: Reactive oxygen species.
MDA: Malondialdehyde.
EL: Electrolyte Leakage.
cDNA: Complementary DNA RNA-Seq RNA sequencing DEGs Differentially expressed genes.
CDSs: CoDing Sequences.
ORF: Open Reading Frame ncRNAs Non-coding RNAs.
DE: Differentially expressed TFs Transcription factors.
BP: Biological processes.
CC: Cellular components.
MF: Molecular functions.
POD: Peroxidase.
SOD: Superoxide Dismutase.
CAT: Catalase.
LEA: Later Embryogenesis Abundant.
ABA: Abscisic acid.
PYR/PYL/RCAR: Pyrabactin Resistance1/Pyr1-Like/Regulatory Components of Aba Receptors.
PP2C: Protein Phosphatase type 2C.
SnRK2: Serine/Threonine Kinases2.
SNF1: Sucrose Non-fermentation 1.
PEG: Polyethylene glycol.
LWP: Leaf water potential.
dNTP: Deoxynucleoside triphosphate.
qRT-PCR: Real-Time Quantitative Reverse Transcription PCR.
