# Supplementary material for: Insight of transcriptional regulators reveals the tolerance mechanism of carpet-grass (Axonopus compressus) against drought
Source: BMC Plant Biol. 2021 Feb 2;21:71. doi: 10.1186/s12870-021-02844-7 (PMC7851936; doi:10.1186/s12870-021-02844-7)
Supplement: Supplementary file 1 — Additional file 1. [file 12870_2021_2844_MOESM1_ESM.docx]

| **ID** | **Gene** | **Primer** | **(5' to 3')** |
| --- | --- | --- | --- |
| 01 | **ABI**4 | ABI4F | CCAAGCTCAACTTCCCGGAG |
| 02 |  | ABI4R | CCTGCGACTCCAGCAGC |
| 03 | **MYB**3 | MYB3F | TAAGAGGCAACCAGTGCTCC |
| 04 |  | MYB3R | GATCAGCGGCAGTGAGGTTA |
| 05 | **MAP kinase**1 | MAP kinaseF | TGCATGGAAATGGCGACCTA |
| 06 |  | MAP kinaseR | CGATATGGCCCCAATCACGA |
| 07 | **NAC** | NACF | CTGCCGGCTCTACAACAAGA |
| 08 |  | NACR | TCCTCCTCTTTGGCCTCCTT |
| 09 | **PIP**2 | PIP2F | ACTGGATCTTCTGGGTTGGC |
| 10 |  | PIP2R | CTCAGCACGAACTGGTGGTA |
| 11 | **WRKY**1 | WRKYF | GTGGTGTACATGGGTGAGCA |
| 12 |  | WRKYR | CGTCGTCGTAGCAGTAGCAT |
| 13 | **Actin** | ActinF | ATGTTGCCATCCAGGCTGTG |
| 14 |  | ActinR | TAAGTCACGTCCAGCGAGGT |

**Table S1:** List of the primers used for the qRT-PCR validation expression analysis of the drought-responsive genes.

**Table S2.** Parameters of sequencing and assembly

| Samples | Raw Reads | Clean Reads | Clean Base(G) | Error Rate(%) | Q20(%) | Q30(%) | GC Content(%) |
| --- | --- | --- | --- | --- | --- | --- | --- |
| Ck-1 | 59966928 | 59356354 | 8.90 | 0.02 | 97.91 | 94.02 | 55.66 |
| Ck-2 | 62246876 | 60952544 | 9.14 | 0.02 | 97.75 | 93.69 | 55.13 |
| Ck-3 | 61106902 | 60154449 | 9.02 | 0.02 | 97.83 | 93.86 | 55.39 |
| DS-1 | 58110216 | 57318402 | 8.60 | 0.02 | 97.61 | 93.29 | 54.94 |
| DS-2 | 56507047 | 55755291 | 8.37 | 0.02 | 97.67 | 93.45 | 55.11 |
| DS-3 | 54903878 | 54192180 | 8.13 | 0.02 | 97.73 | 93.61 | 55.28 |
| Total/Mean | 352841847 | 347729220 | 52.16 | 0.02 | 97.75 | 93.65 | 55.25 |

**Table S3**: DEGs of Transcription factors families of *Axonopus compressus*

| **TF Family** | **up** | **down** | **TF Family** | **up** | **down** |
| --- | --- | --- | --- | --- | --- |
| AP2/ERF-ERF | 62 | 1 | GRF | 1 | 0 |
| B3 | 1 | 4 | HB-BELL | 0 | 2 |
| B3-ARF | 0 | 5 | HB-HD-ZIP | 11 | 0 |
| bHLH | 3 | 2 | HB-other | 4 | 3 |
| bZIP | 19 | 1 | HSF | 24 | 0 |
| C2C2-CO-like | 0 | 1 | LOB | 4 | 0 |
| C2C2-GATA | 1 | 0 | MADS-M-type | 1 | 2 |
| C2C2-LSD | 1 | 1 | MYB | 4 | 0 |
| C2H2 | 14 | 3 | MYB-related | 14 | 4 |
| C3H | 6 | 0 | NAC | 5 | 1 |
| CPP | 1 | 0 | NF-X1 | 1 | 0 |
| DBP | 3 | 0 | RWP-RK | 0 | 1 |
| EIL | 1 | 0 | SBP | 3 | 1 |
| FAR1 | 3 | 9 | TCP | 2 | 0 |
| GARP-ARR-B | 2 | 1 | Tify | 0 | 4 |
| GARP-G2-like | 3 | 5 | TUB | 6 | 1 |
| GRAS | 4 | 1 | WRKY | 13 | 1 |

**Table S4** DEGs of Transcription regulatory factor families of *Axonopus compressus*

| **Transcription regulatory factors families** | **Number** | **Transcription regulatory factors families** | **Number** |
| --- | --- | --- | --- |
| AUX/IAA | 6 | mTERF | 9 |
| DDT | 3 | Others | 6 |
| GNAT | 4 | PHD | 6 |
| HMG | 2 | SET | 11 |
| IWS1 | 2 | SNF2 | 12 |
| Jumonji | 3 | SWI/SNF-BAF60b | 4 |
| LUG | 1 | TAZ | 4 |
| MBF1 | 2 | TRAF | 6 |
