# Supplementary material for: Insight of transcriptional regulators reveals the tolerance mechanism of carpet-grass (Axonopus compressus) against drought
Source: BMC Plant Biol. 2021 Feb 2;21:71. doi: 10.1186/s12870-021-02844-7 (PMC7851936; doi:10.1186/s12870-021-02844-7)
Supplement: Supplementary file 2 — Additional file 2. [file 12870_2021_2844_MOESM2_ESM.docx]

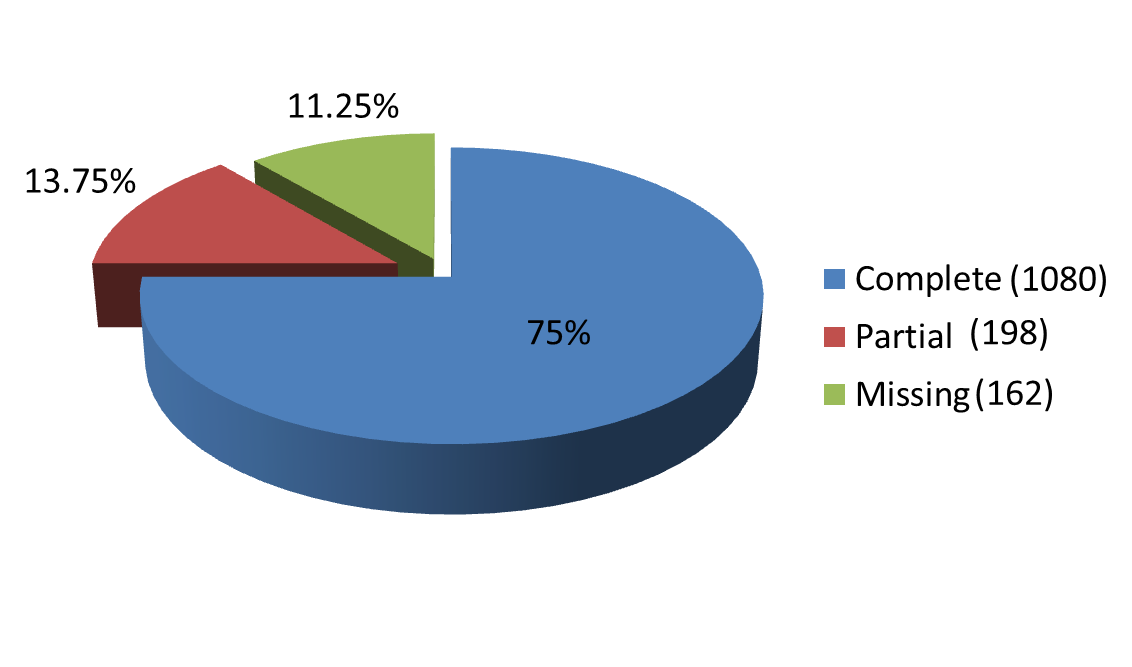


**Figure S1** The genome completement assessments using benchmarking universal single copy orthologs (BUSCO)


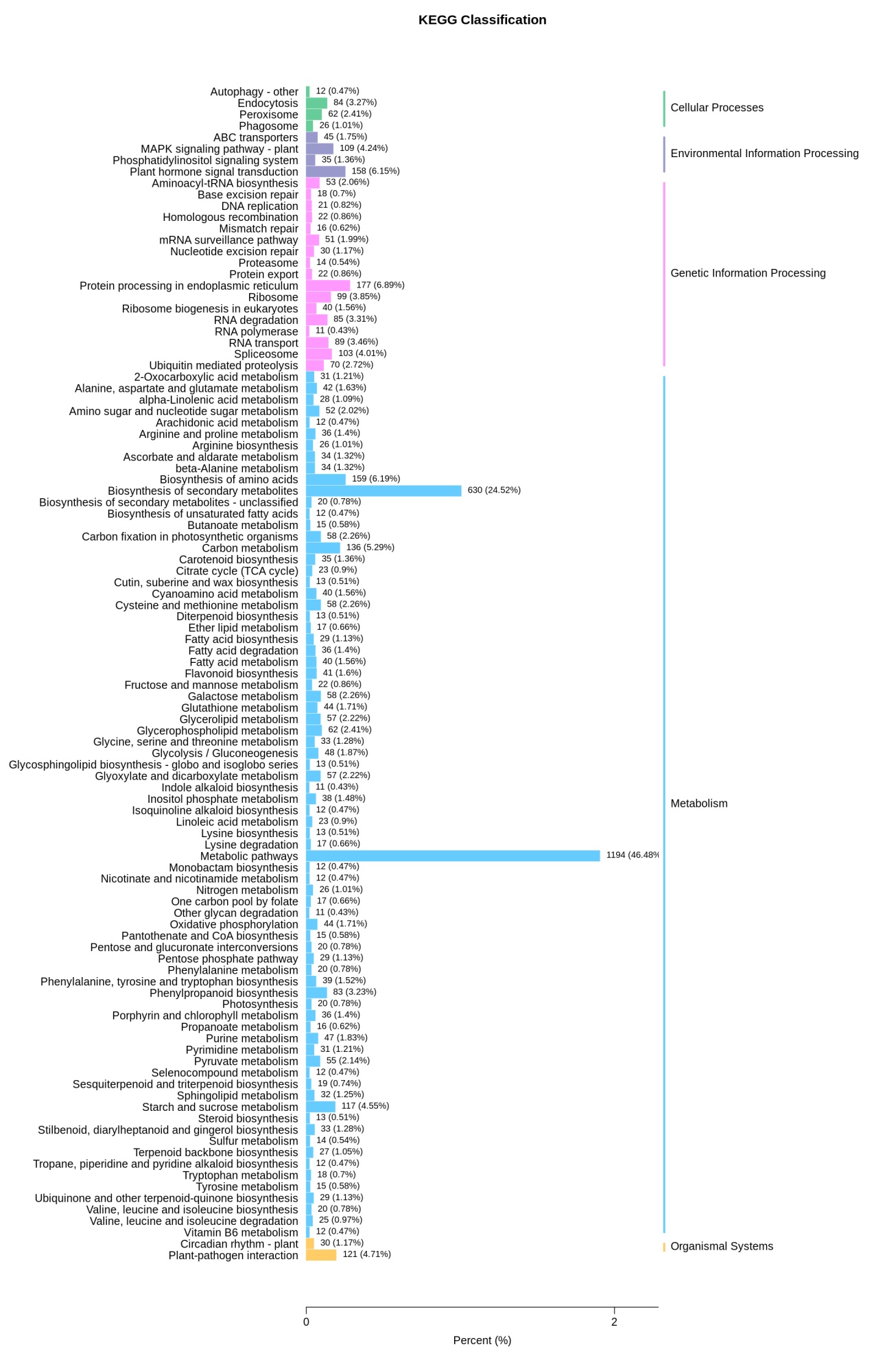


**Figure S2** KEGG pathway annotation for *Axonopus compressus*. The percent of unigenes in each pathway is shown in the small brakets; the pathway categories are shown on the y-axis


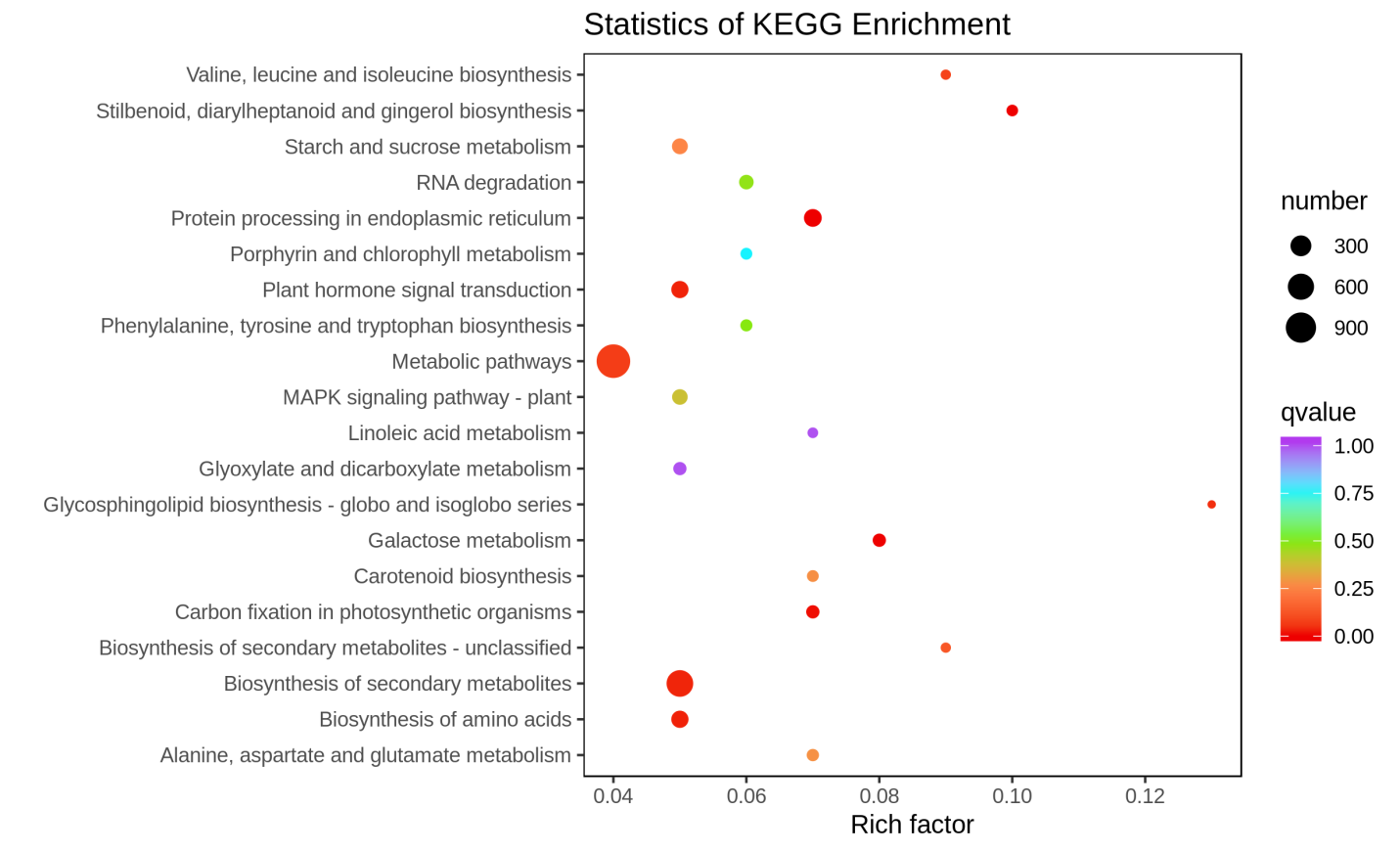


**Figure S3** KEGG pathway enrichment of DEGs in *Axonopus compressus*. The graph shows only the top 20 enriched pathways comparing DS with CK; different colors denotes different Q-Values, and the size of the bubble represents the number of DEGs


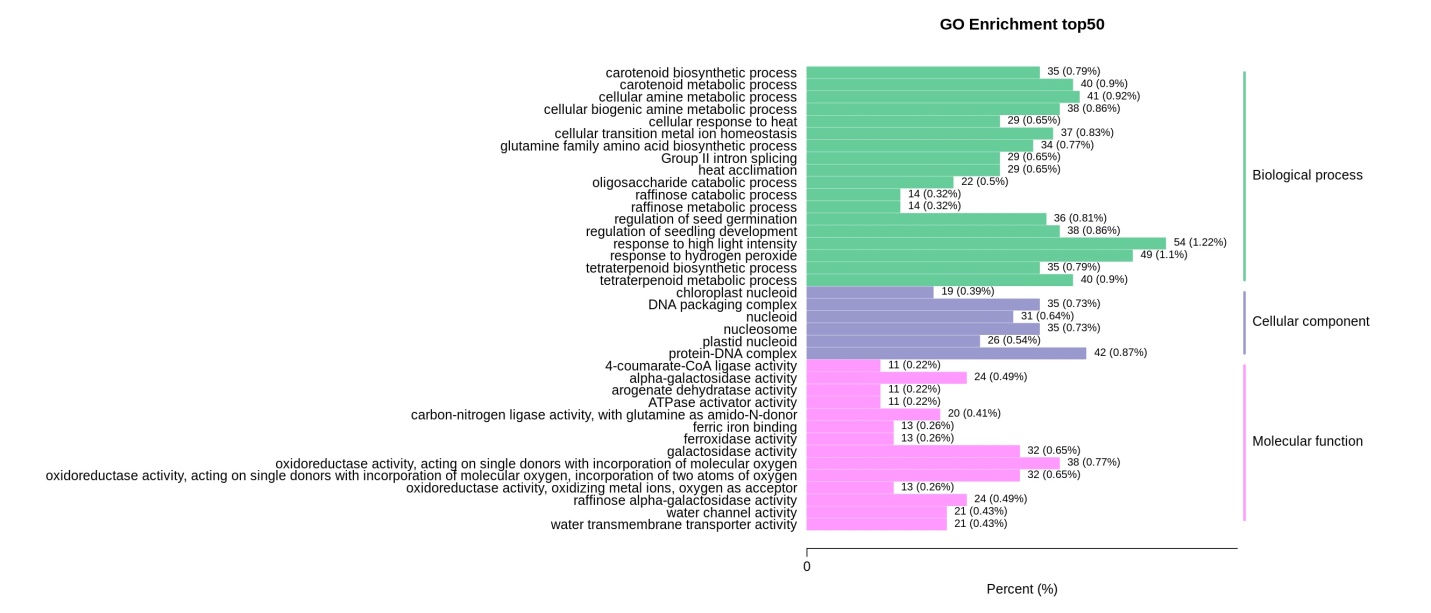


**Figure S4** GO-enriched DEGs in *Axonopus compressus*.
